# 

**Published:** 1916-06-24

**Authors:** 


### CONTENTS.

PAGE

**What is Medical Responsibility?...** ... ... ... 269

**The Doctor's Indifference...** ... ... ... ... 270

**Hospital and Institutional News ...** ... .. 271

The Council Election of the Royal College of Surgeons—Insanity and the Services—Another Medical Conscientious Objector—King Edward VII.'s Hospital, Cardiff—More Zeppelin Hospital Finance—The Greatest Modern Epic—The Latest Anti-Syphilitic Remedies—Advertisements in Voluntary Hospitals—The Sportsmen's Ambulance Fund—Two Richmond War Hospitals—The Royal Liverpool Country Hospital, Heswall—Inquests in War-Time—A Youthful *Gazette*—The Collectors of Herbs—The Wages of a Hospital Labourer—A Home for Defectives at Sheffield—A Sanatorium Project Abandoned—Bristol's New Child-Welfare Scheme—This Week's Drug Market.

**Human Temperaments** ... ... ... ... 277

V.—The Faddist.

**Research and Remedies** ... ... ... ... ... 278

**The School Doctor** ... ... ... ... ... ... 279

The School Doctor and the Tuberculous Child.

**The Lady House Surgeon** ... ... ... ... 280

By an Old-Time Matron.

**Gifts to Hospitals** ... ... ... ... ... ... 280

**The Matrons' and Sisters' Department** ... 281

The Royal British Nurses' Association.—I.

Round the Hospitals.

Guard the Entrance—Hospital Holidays—Nurse-training in War-time—What is the Remedy?—The Cottage Nurse—A Call for Patience.

PAGE

**The Heating of Hospitals ...** ... ... ... ... 283

VIII.—Bristol General Hospital.

**Passing Events and Topics ...** ... ... ... 284

**The Editor's Letter-Box** ... ... ... ... 285

Hospital Nursing in War-Time: Disadvantages of Depleted Staffs—The Compulsory Removal of Phthisical Patients—Children's Country Holidays Fund—National Training School for District Midwives—A Shortage of Catgut.

**What's What Column** ... ... ... ... ... 286

**Medical Appointments** ... ... ... ... ... 286

**Matrons' and Sisters' Appointments** ... ... 286

**Frauds on Soldiers** ... ... ... ... ... ... 286

**The Extensor Response (Babinski's Sign)** ... 275

Its Positive and Negative Value.

**Highland and Islands Medical Service Board** 276

A Policy of Cautiousness.

**THE SPECIAL HOSPITAL SUNDAY SUPPLEMENT** ... ... ... ... ... ... ... 287

**The College of Nursing** ... ... ... ... ... 307

Meeting of Representatives of Nurse-Training Schools.

The Audience.

The Chairman's Speech.

The Discussion.

**The Institutional Worker Section** ... ... 1

The Nursing Organisation of a Children's Open-air Ward.

Relative Cost of Provisions.

Enquiries and Answers.

